# Physicians’ experiences and actions in making complex level-of-care decisions during acute situations within older patients’ homes: a critical incident study

**DOI:** 10.1186/s12877-023-04037-3

**Published:** 2023-05-24

**Authors:** Elin-Sofie Forsgärde, Mattias Rööst, Carina Elmqvist, Bengt Fridlund, Anders Svensson

**Affiliations:** 1 Department of Health and Caring Sciences, Linnaeus University, PO Box 451, Växjö, 351 95 Sweden; 2 Centre of Interprofessional Collaboration within Emergency Care (CICE), Linnaeus University, Region Kronoberg, PO Box 1207, 351 95, 352 54 Växjö, Växjö, Sweden; 3 Ambulance Service, Region Kronoberg, PO Box 1207, 351 95, 352 54 Växjö, Växjö, Sweden; 4 Department for Research and Development, Region Kronoberg, PO Box 1223, 351 12 Växjö, Sweden; 5 Department of Clinical Sciences in Malmö, Family Medicine, Lund University, PO Box 50332, 202 13 Malmö, Sweden

**Keywords:** Aged, Ambulatory care, Decision-making, Physicians, Qualitative research

## Abstract

**Background:**

Complex level-of-care decisions involve uncertainty in which decisions are beneficial for older patients. Knowledge of physicians’ decision-making during acute situations in older patients’ homes is limited. Therefore, this study aimed to describe physicians’ experiences and actions in making complex level-of-care decisions during the assessment of older patients in acute situations within their own homes.

**Methods:**

Individual interviews and analyses were performed according to the critical incident technique (CIT). In total, 14 physicians from Sweden were included.

**Results:**

In making complex level-of-care decisions, physicians experienced collaborating with and including older patients, significant others and health care professionals to be essential for making individualized decisions regarding the patients’ and their significant others’ needs. During decision-making, physicians experienced difficulties when doubt or collaborative obstructions occurred. Physicians’ actions involved searching for an understanding of older patients’ and their significant others’ wishes and needs, considering their unique conditions, guiding them, and adjusting care according to their wishes. Actions further involved promoting collaboration and reaching a consensus with all persons involved.

**Conclusion:**

Physicians strive to individualize complex level-of-care decisions based on older patients’ and their significant others’ wishes and needs. Furthermore, individualized decisions depend on successful collaboration and consensus among older patients, their significant others and other health care professionals. Therefore, to facilitate individualized level-of-care decisions, the health care organizations need to support physicians when they are making individualized decisions, provide sufficient resources and promote 24 − 7 collaboration between organizations and health care professionals.

## Introduction

Redesigning health care organizations to meet the needs of an aging population and extending the care delivered within older patients’ homes is an ongoing global discussion [1–3]. Historically, in Sweden, community health nurses (CHNs) and general practice (GPs) specialists [4] or ambulance personnel [5] have conducted assessments of older patients within their homes. In recent years, CHNs and GP specialists have performed the majority of home visits. Nevertheless, there has been a rapid increase in ambulatory teams and physicians from other healthcare organizations providing home visits for older patients in acute situations [6], but knowledge of this increase is limited. The increase is possibly explained by the lack of GP specialists and their limited time to make home visits [4].

In the UK, physicians describe assessing older patients in acute situations within their homes with limited diagnostic equipment. Following this evaluation, they determine the necessary treatment and the appropriate level of care [7]. This circumstance is similar in Sweden. Older patients in acute situations suffer from acute diseases, the worsening of chronic diseases or injuries [8]. Making level-of-care decisions for older patients, such as if treatment should be provided at home, in a primary health care (PHC) centre or in a hospital, is complex due to age-related physiological changes, comorbidities and polypharmacy. Furthermore, older patients have an increased risk of presenting with non-specific symptoms [9] and frailty [10]. According to a study conducted in the USA, the leading cause of death among older trauma patients is not the injury itself but rather the combination of the injury, previous diseases and frailty [11]. Complex decisions, such as level-of-care decisions for older patients, include multiple interacting parts, while not all parts are known; furthermore, they are nonlinear with low certainty of the outcome [12]. These interacting parts may include the following: patients’ needs and wishes regarding care, the physicians’ experiences and knowledge of the advantages and disadvantages of different levels of care, and accessibility to different levels of care and diagnostic possibilities [7]. Complex level-of-care decisions present risk factors [13], such as the deterioration of older patients’ conditions [14] and missed or delayed diagnoses resulting in adverse outcomes [15–17]. Physicians worldwide describe making level-of-care decisions for older patients in acute situations as potentially demanding and connected to the uncertainty of overlooking severe illness [7, 18, 19]. Furthermore, physicians perceive the uncertainty of underlying causes and the older patients’ need to receive adequate treatment at hospitals as reasonable grounds for making a decision for hospital care [19]. According to a study conducted in the USA, physicians report that they often have the final word in the decisions that are made but that they prefer shared decision-making with older patients [20]. Knowledge of complex level-of-care decision-making for older patients within their homes is limited. Furthermore, the complexity and risk in making level-of-care decisions for older patients highlight the need for further studies [21].

### Aim

The aim was to describe physicians’ experiences and actions in making complex level-of-care decisions during the assessment of older patients in acute situations within their own homes.

### Method

The study was performed with an inductive descriptive design and analyzed with the critical incident technique (CIT). The CIT is used to analyze human behavior (experiences and actions) in well-defined incidents through, for example, interviews [22]. Critical incidents included descriptions of situations, from the beginning to the end, holding complex level-of-care decisions that participants experienced as significant [23]. A critical incident started when a physician gained information about the acute situation and ended when a level-of-care decision was made.

#### Setting

The study was conducted in different health care regions and municipalities in Sweden, which has publicly funded health care and approximately 10 230 000 inhabitants; of these inhabitants approximately 20% are aged 65 years or older. Health care regions and municipalities share responsibility for health care [24], however, the capital is an exception, where the health care region has total responsibility. Physicians working in ambulatory teams have various specializations, such as GP specialist, or emergency care; and they belong to various parts of the health care system, such as primary health care (PHC) centers or hospitals. Furthermore, they conduct home visits to assess older patients in acute situations [6]. Physicians have 5 ½ years of higher education, 1–1 ½ years of a clinical internship, and an additional 5 years of clinical specialization residency experience. Physicians assessing older patients within their homes either work on a team with a registered nurse (RN) or alone and frequently cooperate with CHNs. CHNs include RNs and RNs with various specializations working within home health care and at nursing homes. CHNs must complete three years of higher education to become an RN and an additional 1–1 1/2 years of specialization within higher education. Moreover, within home health care and nursing home work, assistant nurses must have one to two years of secondary school education and there is no required care education for care assistants. Accordingly, when physicians refer to health care professionals, several types of professionals are included.

#### Participants and data collection

The inclusion criterion was physicians with experiences in making complex level-of-care decisions during acute situations within older patients’ homes. The exclusion criterion was physicians without the previously described experience.

The sample of 14 physicians from 13 different ambulatory care organizations was strategically selected to encompass a variety of sociodemographic and professional characteristics, (shown in Table 1), as well as healthcare organizations and geographical locations, in accordance with the six health care regions in Sweden. One to five participants were included from each health care region. Approval to conduct the study was obtained from operations managers in the healthcare organization that performed assessments within older patients’ homes. The operations managers thereafter sent an information letter regarding the study to the physicians, who in turn sent approval of participation to the first author without the operations managers’ knowledge. The first author contacted the participants through email and scheduled the interview date and time and asked if they wanted to conduct the interview over Zoom or via telephone. All participants preferred telephone interviews. A document with the study information and consent to participate were sent by mail. The participants answered the questions and returned the signed document of consent to participate to the first author by mail.

**Table 1:** Socio-demographic and professional characteristics of physicians included in the study (N = 14)

	*Number*
*Sex*	
Female	6
Male	8
*Age*	
40–50	9
51–60	1
61–70	2
71–80	2
*Years as physician*	
10–20	9
21–30	3
31–40	1
41–50	1
*Years of assessing patients within their homes*	
1–10	10
11–20	1
21–30	1
31–40	2
*Specialization residency*	
Emergency medicine	1
General practice	7
Geriatrics	1
≥ 2 specializations, within: general practice, internal medicine, emergency care	5

The semistructured telephone interviews were conducted between August 2020 and October 2021. Prior to the interview, information from the study was repeated for the participants, they were queried as to whether the interviews could be tape-recorded which all agreed upon. Furthermore, a definition of ‘complex level-of-care decisions’ was given: “*When a level-of-care decision is not obvious, and different aspects need to be weighed against each other*” [25]. During the interview, the concept “critical incidents” was not used; instead, the participants were encouraged to describe two or three situations that included complex level-of-care decisions. The nine CIT interview questions developed by Fridlund et al. [23] to facilitate the adaption of CIT to healthcare sciences were asked to gain detailed descriptions from different perspectives on experiences and actions connected to the study aim (Table 2). Follow-up questions were further asked to gain more information and clarify the descriptions, such as *“Can you please describe that more?”*. Following the initial interview, the research questions were considered to ascertain whether the participant had understood them in the specific study context. The participant understood the questions and no changes were deemed necessary. The interviews lasted between 50 and 75 minutes. Each critical incident, in turn, comprised several experiences and actions, with 450 in total. Finally, the interviews were transcribed verbatim by the first author.

**Table 2:** Description of the nine critical incident technique (CIT) interview questions [23]

1. Can you, please, describe the environment in which the care decision was made? 2. Can you, please, in detail, describe what happened? 3. What made the care decision complex? 4. How did you act in connection to the care decision? 5. What was your mindset in the situation? 6. What did you think during and afterwards? 7. What did you feel during and afterwards? 8. What were the most challenging aspects of the care level decision? 9. What has this situation meant for you afterwards?	

### Data analysis

The structural analysis began by reading the interview text several times to become familiar with its content. Experiences and actions while making complex level-of-care decisions were marked with numbers, extracted from the text, condensed and labeled to facilitate further analysis [23]. The experiences and actions were sorted and analyzed separately. The analysis process was the same for both parts [23]. Similar experiences were grouped together at a descriptive level to compile the quotations, which resulted in 16 subcategories. Similar subcategories were then grouped and abstracted into six categories, and the categories were further clustered and abstracted into two main areas (Table 3). The actions comprised 15 subcategories, six categories and two main areas (Table 4).

**Table 3:** An overview of main areas, categories and sub-categories of physicians’ experiences of making complex level-of-care decisions

Sub-categories *(n)*	Categories	Main areas
-Want to include patients’ wishes *(5)* -Patients and significant others need clear information *(5)* -Want to find the ”best” solution for patients *(14)* -Being within patients’ homes facilitates the understanding (13) -Being open to reconsidering decisions *(9)* -Collaborate with healthcare professionals helps *(14)* -Significant others as available resources *(14)* -Advantages of relational continuity *(20)*	The need for inclusion The wish to individualize decisions The need for collaborative support	Collaboration essential for adaptation
-Doubt one´s ability *(5)* -Feel unsure in treatments and decisions *(12)* -Feel frustrated when patients’ refuse care *(18)* -Disagree with patients and significant others *(19)* -Collaborate with healthcare professionals are complex *(15)* -Unclear decision basis *(10)* -Feel alone in decisions *(6)* -Lack of resources *(20)*	The doubt within decisions The sense of obstructed collaboration The feeling of being exposed	Difficulties during decision-making

**Table 4:** An overview of main areas, categories and sub-categories of physicians’ actions while making complex level-of-care decisions

Sub-categories *(n)*	Categories	Main areas
-Allowing visits to take time *(15)* -Listening to patients and significant others *(17)* -Performing individualized assessments *(31)* -Considering the short remaining life expectancy *(11)* -Weighing pros and cons *(11)* -Following patients’ and significant others’ wishes *(28)* -Accepting patients’ wishes *(7)* -Leading patients and significant others right *(25)* -Providing straightforward and clear information *(23)*	Searching for understanding Considering unique conditions Adjusting to wishes Guiding through knowledge	Adaptation pursuits
-Collaborate with involved healthcare professionals *(21)* -Arranging follow-up care *(18)* -Seeking consultation *(11)* -Seeking consensus with patients and significant others *(5)* -Seeking consensus with healthcare professionals *(12)* -Seeking alternative solutions *(16)*	Promoting collaboration Striving for consensus	Collaborative endeavors

## Results

### Physicians’ experiences in making complex level-of-care decisions

The physicians’ experiences in making complex level-of-care decisions during the assessment of older patients within their homes were described in two main areas: ‘Collaboration essential for adaptation’ and ‘Difficulties during decision-making’.

### Collaboration essential for adaptation

During complex level-of-care decisions, physicians experienced collaboration with all involved, including older patients, their significant others, and various healthcare professionals, to be essential in adapting care to the patient’s needs. This was experienced through the need for inclusion, the wish to individualize decisions and the need for collaborative support.

### The need for inclusion

While making complex level-of-care decisions, physicians experienced including older patients and their significant others in a dialog by giving clear information to be an essential prerequisite for understanding the situation and expectations. Physicians experienced their role as being supportive in older patients’ and their significant others’ decision-making. The participation of older patients and their significant others facilitated decision-making; simultaneously, difficulty existed in knowing if they understood the consequences of their decisions: *“It is difficult to know if the patient has the ability to understand the consequences of his decision to forgo hospital care.” –Physician 2*.

### The wish to individualize decisions

While making complex level-of-care decisions, physicians wished to identify older patients’ needs for making individualized decisions. Knowing the older patients’ previous experiences and wishes regarding their care simplified these decisions. Complex level-of-care decisions were not based on standardized decision support but rather on designing the care for the unique older patient: *“It is important to see who the patient is and where she is in her illness and life when deciding on the level of care.” –Physician 8.*

Making individualized decisions for older patients was facilitated by being within their homes due to physicians having sufficient time for dialogs, trust-building and receiving information from multiple sources for a comprehensive understanding: *“Meeting the patient within the home gives a good contact and picture of how he manages, which surpasses all other information and makes it easier to make a reasonable decision” – Physician 11.* Furthermore, it was experienced as hard but necessary to reconsider one’s own decisions and change the directions of previous care plans in each situation to continuously make individualized decisions.

### The need for collaborative support

While making complex level-of-care decisions, physicians experienced support through collaboration with older patients, their significant others and their healthcare professionals. Trust facilitated collaboration and decision-making and was established through regular encounters with older patients, their significant others and their healthcare professionals: “*The nurse at the nursing home trusted me because I had already been there before, and she had been able to call me whenever she needed to. So, there was no problem in handing over a lot of palliative prescriptions and such”–Physician 12*. Consensus among all persons involved and having an organization with sufficient resources was experienced as essential for collaborative support while making complex level-of-care decisions: *“The treatment involved a great effort by CHNs in providing medicine 24 − 7 within the patient’s home, but they knew the situation and were willing to try.” –Physician 4.*

Collaborative support was achieved by significant others acting as older patients’ representatives when they were unable to care for themselves and managing the care within the homes 24 − 7. Significant others’ abilities to support older patients within their homes were considered when making decisions: *“It is complex to assess the patient’s needs and what the significant other is able to and wants to do.” –Physician 10.*

Collaborative support was achieved by CHNs when they had previous knowledge of a unique older patient. A lack of continuity was experienced as making decision-making difficult due to increasing the risk of deciding on disproportionate care: *“It is difficult not knowing the patient, not knowing the patient’s usual status and how long the deterioration has lasted; is it something temporary and curable?” –Physician 6.* However, successful collaboration with CHNs was experienced as being bound to specific persons. Collaborative support was further achieved while making complex level-of-care decisions by contacting physicians at hospitals due to the ability to discuss possible medical options.

### Difficulties during decision-making

During complex level-of-care decisions, physicians experienced difficulties when doubt existed within decisions, collaboration was obstructed and when feeling exposed.

### The doubt within decisions

While making complex level-of-care decisions, physicians doubted themselves due to a lack of experience in making certain medical decisions. Physicians experienced uncertainty in their decisions regarding performing enough or too many diagnostic tests and if other treatments were possible: *“It is a difficult approach to sometimes not doing anything medically because it is ethically correct and to sometimes investigate even though the patient is seriously injured.” –Physician 2.* Uncertainty further arose when physician did not know if the treatment was sufficient until afterward: *“I wondered if the decision was right, if the patient became more alert and started eating again; was it only a temporary improvement and a postponement of death? The decision was not obvious.” –Physician 6.*

### The sense of obstructed collaboration

While making complex level-of-care decisions, difficulties in collaboration were experienced as obstructive due to collaboration being essential for the older patients’ care.

A lack of consensus in level-of-care decisions among patients, significant others, physicians and other health care professionals obstructed collaboration and was experienced as demanding. A lack of consensus between older patients and their significant others was experienced based on different degrees of preparedness. Frustration arose when older patients refused assessment or the needed care, especially when they risked dying accordingly: *“It is frustrating when a patient has a curable condition but does not want to be cared for in hospitals and the treatment is unable to be given at home.” –Physician 8.*

The existence of doubts within collaboration between health care professionals was experienced as problematic when nonstandard medical treatment decisions were made: *“When you make decisions that are little outside the box and someone in the group panics, the whole group follows, and then the patient usually ends up at a hospital.” –Physician 9.* Furthermore, collaboration difficulties occurred when several physicians with various specializations were involved in the older patients’ care and only performed the work they thought was their responsibility.

### The feeling of being exposed

While making complex level-of-care decisions, physicians felt exposed when they needed to balance what was reasonable to suspect and what to exclude, especially when an older patient had non-specific symptoms. Physicians experienced it identifying unusual life-threatening diagnoses to be essential. Diagnostic possibilities were, however, limited within the homes at the same time, as all assessments and diagnostics strained the older patients. It was experienced as a more demanding decision to care for older patients within their homes compared to referring them to hospitals. The physicians, however, took this responsibility when judged as optimal due to possible risks of hospital care: *“By sending the patient to another care level, the responsibility is removed from you… I try to take that responsibility and communicate it to the patient; I think that the risk of being at a hospital is underestimated.” –Physician 10*.

Feeling exposed was further connected to experiences of loneliness in making decisions when being pressured by other health care professionals to resolve situations even if a resolution was not possible. Physicians experienced a sense of failure when the outcomes were not optimal for older patients but also a need to forgive themselves for not always making optimal decisions: *“Sometimes you think, why did I do that, but then…I may have to forgive myself because you cannot always act razor-sharp, you fumble a little sometimes…and tread water, and then come to the decision you want to make.” –Physician 3.*

Moreover, physicians experienced feeling exposed and betraying older patients when they were unable to meet their care needs due to organization limitations: *“We do not have an organization that is adapted to take care of patients who become acutely ill during an on-call time, neither physicians nor other health care professionals.” –Physician 8.*

### Physicians’ actions while making complex level-of-care decisions

While making complex level-of-care decisions, physicians’ actions were described in two main areas: “Adaptation pursuits” and “Collaborative endeavors”.

### Adaptation pursuits

During complex level-of-care decisions, physicians took action to pursue the adaption of care for the older patient; by searching for an understanding, considering unique conditions, adjusting to wishes and guiding through knowledge.

### Searching for understanding

While making complex level-of-care decisions, physicians took a sufficient amount of time to search for an understanding of older patients’ situations: *“It is important to not go too fast, not making decisions on too little information.” –Physician 7.* Taking time was further connected to being patient and ensuring that trust would grow in the encounter.

To understand, physicians listened to older patients’ and their significant others’ descriptions of their situations, which became the basis for making decisions. Decisions were based both on older patients’ and their significant others’ needs, which were described as especially important during care at the end of life. Physicians worked to actively involve older patients and their significant others during the decision-making process: *“It is important to listen to the patient; he was so affected by the disease that you easily could have run over him.” –Physician 9.* Individualized assessments further increased understanding, such as making structural assessments, taking blood samples and performing ultrasounds. Judgment of deteriorations and possibilities to manage their daily life were then added to the grounds for the decision ground: *“It is so individual, it is not possible to follow a template in these decisions…it´s the experience template and the information from the patient’s medical record that are used.” –Physician 6.*

### Considering unique conditions

While making complex level-of-care decisions, physicians considered unique conditions regarding an older patient’s remaining life expectancy by identifying where they were in life and within the disease course. The goal of level-of-care decisions was to optimize comprehensive well-being and decrease suffering: “*The treatment goal was that she would feel good rather than keep the blood sugar at an exact level.” –Physician 2*.

Physicians weighed the pros and cons of different levels of care to determine an optimal decision for a unique older patient. It was, however, difficult to balance existing treatment options, risks and older patients’ wishes: *“We considered, if the patient went to the hospital… the treatment would be faster, but there would be a delay by sending him there, and a risk that he couldn´t handle the stress.” –Physician 9.*

### Adjusting to wishes

While making complex level-of-care decisions, physicians adjusted care accordingly to older patients’ and their significant others’ wishes. The wishes weighed heavier in the decisions than previously written care plans: *“The patient had clearly decided to receive care at home at the end of her life, but she was afraid and changed her mind when she was affected by breathing difficulties. No one can know that in advance, so the care plans need to be reconsidered.” –Physician 8.*

Accepting older patients’ wishes that contradicted physicians’ suggested care was followed by knowing that older patients and their significant others understood the consequences of their decision: “*They want it as they have it, and then you have to accept it… and meet them where they are.” –Physician 2.* When older patients did not want the recommended care, their significant others were informed, and the decision was noted in the patients’ medical records. The older patients were further informed of being free to change their wishes whenever they wanted: *“Physicians cannot forget that the decisions must be fresh and that the patient must always be able to regret or change their decisions made.” –Physician 14.*

### Guiding through knowledge

While making complex level-of-care decisions, physicians worked proactively to gain knowledge of older patients’ and their significant others’ understanding of the situation and the basis of their wishes. Furthermore, when wishes were based on unawareness or were disadvantageous for older patients, physicians tried to guide them through knowledge: *“I do it because I’m convinced that this is what they truly want; they do not understand what they are turning down, and it is my job to help them.” –Physician 4.* Physicians explained why a decision was reasonable with facts and strived to provide straightforward and clear information emphatically, emphasizing avoiding misunderstandings. The given information concerned descriptions of older patients’ situations, recommendations and risks: *“I recommend that you go to the hospital; if you don´t go, there is a risk of death.” –Physician 14.* When older patients and their significant others trusted physicians due to relation continuity, the physicians were more straightforward, and the motivation was experienced as having a larger impact.

### Collaborative endeavors

During complex level-of-care decisions, physicians took action to foster collaboration with all involved; by promoting collaboration and striving for consensus.

### Promoting collaboration

While making complex level-of-care decisions, physicians collaborated with older patients, their significant others and other health care professionals. At times, physicians’ collaboration with significant others enabled older patients to accept receiving the suggested care. At other times, when the collaboration was obstructed, physicians needed to stand up against significant others or other health care professionals to protect the older patients’ wishes: *“I’m not sure if the daughter was happy. Maybe she felt pressured in the decision, but in the situation, there was no other ethical moral alternative than to do as the patient wanted…” –Physician 4.*

Information about older patients, their significant others and their care situation was gained through collaboration with CHNs. CHNs further arranged and provided the care needed: *“I work with CHNs, and we talk before and after (the meeting with the older patient) regarding if there is something special we need to think about…so we support each other.” –Physician 5.* Follow-up care was provided in collaboration with CHNs and assistant nurses to evaluate the decisions that were made. At times, other healthcare organizations were contacted to ensure and continue follow-up care. Collaboration also included consultation with colleagues or physicians at hospitals for support in making decisions: *“I often pick up the phone to call (physicians at the hospital), and there is never anyone that says no… I believe that good health care is teamwork.” – Physician 13.*

### Striving for consensus

While making complex level-of-care decisions, physicians strived to reach a consensus with older patients, their significant others and their health care professionals. By working together, a common consultation was made about how the situation should be solved: *“My way of dealing with this is to work together, to collaborate with everyone involved, how do we solve this…” –Physician 13.* Physicians sought to reach a consensus by reasoning with CHNs to reconcile the situation and discuss options for managing medical treatment at home. Compromises were searched for to fulfill the wishes of older patients and meet their medical needs. The goal was to manage situations as ideally as possible by making compromises, so everyone felt safe and in control. If a consensus was not reached, the conversation was resumed on another day, when everyone had time to think about the situation: *“How can we know a 100% that the patient is in a palliative phase? At times, we try fluid and antibiotics… to buy time in the discussion for achieving a consensus.” –Physician 7*, and *“…and they thought their mother should be given antibiotics, but we thought the patient was most likely at the end of her life,… but I told them that we could try antibiotics for a couple of days to see if the situation would turn around” –Physician 1.* When consensus was not reached at all, physicians searched for other solutions, such as providing medical treatment, that usually given in hospitals at home, or avoiding ED visits by admitting older patients directly to hospital wards: *“She did not want to be there (at the hospital), she was extremely anxious; the only decision we could make was to try to treat her at home…but if it did not succeed, she would die…” –Physician 4.*

## Discussion

When making complex level-of-care decisions during acute situations within older patients’ homes, physicians experienced collaboration with all persons involved, including older patients, their significant others, and other healthcare professionals, as essential for individualizing decisions for the older patients and their significant others. A feeling of being lonely and exposed in decision-making arose when physicians were unsure, collaboration obstruction occurred, or resources were lacking. Physicians’ actions when making complex level-of-care decisions consisted of searching for an understanding of a unique older patients’ life expectancy, wishes and needs, and their significant others’ wishes and needs. Furthermore, physicians guided patients towards perceived beneficial decisions and searched for a consensus with all persons involved. Moreover, physicians accepted older patients and significant others will to not follow the suggested care when judging their understanding of the consequences. Consequently, the following discussion focuses on individualizing decisions and the need for collaboration in making complex level-of-care decisions for older patients in acute situations.

The results show that physicians experiences a need for individualizing decisions for older patients and take action to this end by identifying the wishes and needs of older patients and their significant others and balancing treatment options and risks. These experiences and actions in acute situations are similar to other studies in planned primary care, which highlights the need for a comprehensive understanding of patients within their contexts to decrease uncertainty when making individualized decisions [12, 26, 27]. However, this study adds to the complexity of assessing the ability of significant others to care for the older patient at home. Complex theories guide GP specialists in complex situations and are described as multidimensional and nonlinear, and the patient is interconnected to others [27], while changes in one part affects another [12]. This standpoint broadens the medical traditions of identifying specific conditions to instead determine older patients’ unique circumstances and experiences of their illnesses as grounds for decision-making [27]. The results show that physicians individualize decisions in each encounter to ensure that the care is beneficial for an older patient. However, individualized decisions vary, which makes clinical practice guidelines not always possible or preferable to follow [28, 29]. Standardizing health care has positive effects in some areas but contradicts older patients’ needs for individualized care [28, 29]. Consequently, a comprehensive understanding of older patients and their significant others is valuable in providing individualized care for older patients in acute situations. Physicians need to be supported by their health care organizations and respected by other health care professionals when making decisions that focus on individualization rather than on following clinical practice guidelines and previous decisions.

The results show that physicians experiences a need for collaborative support and take action to foster support to individualize complex level-of-care decisions. These experiences and actions are similar to another study in nursing homes, where physicians describe a dependency on well-functioning collaboration with other health care professionals and act proactively to ensure everyone is aligned [33]. Complex level-of-care decisions affect older patients, their significant others and the health care professionals involved [12], and ethical decision-making ensures that all perspectives are considered [30]. Consensus is a ‘reasonable deliberation’ that involves and respects the perspectives all persons involved [31]. Consensus between older patients and physicians has been described as important in previous studies [32, 33]. However, the results show that decision-making is difficult when a consensus is not achieved and the ethical principles of beneficence and patients’ autonomy are conflicting [34]. Beneficence is related to decisions that are judged as beneficial for older patients, and autonomy is related to patients’ wishes [34]. Joint deliberations enable an understanding of others’ perspectives, and one’s own position becomes transparent to others, allowing relevant considerations to be brought forward, such as facts, interests, and perspectives [31]. The results show that physicians use joint deliberations by discussing with all persons involved and, for example, finding out if older patients and their significant others are aware of the consequences of their decisions. This result is similar to another study [35]. When older patients have decreased decision competence or disagree with physicians, a beneficial approach is trying to influence them to make decisions that are perceived as the most beneficial, which possibly results in a compromise [36]. Older patients’ rights to informed consent involve the right to be informed of risks and the right to autonomy [34]. The results show that physicians guide older patients and their significant others by emphatically providing straightforward and clear information. If possible, the discussion is resumed another day for all persons involved to have time to reconsider the situation. If consensus is not reached and older patients and their significant others are judged to be aware of the consequences of their decisions, physicians respect their autonomy. Ethical principles and patients’ rights are considered helpful when making complex level-of-care decisions [34]. Subsequently, collaboration supports complex level-of-care decisions by reaching a consensus among older patients, their significant others and the health care professionals involved. When a consensus is not reached, actions are taken to provide information, give time, make compromises and accept older patients’ decisions.

### Methodological considerations

The trustworthiness of the study was reached by considering the concepts of credibility, dependability, confirmability and transferability throughout the research process. The study´s credibility was strengthened by including participants with experiences of making complex level-of-care decisions, and the critical incidences were described in detail by the participants and included 450 experiences and actions [22, 23]. The sample size was guided by information power, meaning that the high amount of relevant information indicated that a lower number of participants was sufficient [37]. An aspect affecting the sample size was the difficulty in finding physicians who wanted or had the ability to participate in the study; this was possibly affected by the high workload due to the ongoing COVID-19 pandemic. The inclusion of more participants would possibly add additional variations of experiences and actions. However, the goal was not to reach a total inclusion of all experiences or actions of the studied phenomenon [37]. The goal was instead to include well-targeted participants with situational variances [37]. The study’s dependability was strengthened due to the participants’ situational variances [23]. The time between the situation and the interview is crucial because the memory of an event becomes less reliable with time. However, the detailed descriptions revealed that the participants had a clear memory of the situation. The participants made their own choices of which situations to describe, which increased the chance that they chose a situation that was memorable. An identified risk was that the participants may have only described critical incidents with positive outcomes. However, critical incidents with negative outcomes were also described, ensuring that even these aspects were included. A way of decreasing this risk would have been to ask the participants to describe critical incidents with positive and negative outcomes [23]. The choice to define the concept of complex care decisions, instead of giving an example from another context as recommended in the literature [23], was to limit the impact on the participants’ described situations, which possibly strengthened the confirmability of the study. The confirmability was further strengthened due to the first and last authors conducting the analysis together, and the result description was discussed with all authors to handle biases and reflect upon the authors’ understanding to avoid affecting the results [23, 38]. The results are assumed to be possibly transferable to decision-making in other care contexts.

### Conclusions and implications

The results show that physicians’ experiences and actions during acute situations in older patients’ homes involved a need to collaborate with all persons involved to make individualized decisions for older patients and their significant others. Physicians experienced being lonely and exposed when they were unsure, difficulties in collaboration occurred, or resources were lacking. Physicians’ actions involved comprehensively understanding older patients’ and their significant others’ wishes and needs. Through knowledge, physicians guided older patients toward beneficial decisions and aimed to reach a consensus on the decision with all persons involved. Physicians’ actions also involved accepting older patients’ and their significant others’ wishes when contradicting the suggested care if they seemed to understand the consequences.

The results highlight the difficulties in making complex level-of-care decisions and that the comprehensive understanding of older patients and their significant others and collaboration with all persons involved are valuable. At individual level, considering ethical principles, patient rights, and conflicts between them possibly provides support when making complex level-of-care decisions. At a group level, including older patients and their significant others in level-of-care decisions possibly facilitates the understanding of wishes and needs and supports individualized decisions. All health care professionals need to be aware of the importance of contributing to collaboration to enable individualized level-of-care decisions. Reaching a consensus is essential in individualized decision-making and is facilitated through dialogs and sufficient time; for older patients, their significant others and all health care professionals to be comfortable with the decision. At an organizational level, support from health care organizations and other health care professionals is needed when level-of-care decisions that are individualized for an older patient diverge from general clinical guidelines. Furthermore, sufficient resources and collaboration between organizations and various health care professionals must be extended to provide individualized 24 − 7 care. At an educational level, a comprehensive understanding of patients and their significant others, collaboration, consensus, ethical principles and patients’ rights should be considered valuable in making complex care level-of-care decisions for older patients in acute situations.

## Data Availability

The dataset analysed during the current study are not publicly available due to this authorization has not been included in the Swedish Ethical Review Authority’s ethical approvement of the study, but are available from the corresponding author on reasonable request.
